# Policy-Gradient and Actor-Critic Based State Representation Learning for Safe Driving of Autonomous Vehicles

**DOI:** 10.3390/s20215991

**Published:** 2020-10-22

**Authors:** Abhishek Gupta, Ahmed Shaharyar Khwaja, Alagan Anpalagan, Ling Guan, Bala Venkatesh

**Affiliations:** Department of Electrical, Computer and Biomedical Engineering, Ryerson University, Toronto, ON M5B2K3, Canada; abhishek1.gupta@ryerson.ca (A.G.); Akhwaja@ryerson.ca (A.S.K.); lguan@ee.ryerson.ca (L.G.); bala@ryerson.ca (B.V.)

**Keywords:** state representation learning, variational auto encoder, deep deterministic policy gradient, soft actor-critic, autonomous driving, Markov decision process

## Abstract

In this paper, we propose an environment perception framework for autonomous driving using state representation learning (SRL). Unlike existing Q-learning based methods for efficient environment perception and object detection, our proposed method takes the learning loss into account under deterministic as well as stochastic policy gradient. Through a combination of variational autoencoder (VAE), deep deterministic policy gradient (DDPG), and soft actor-critic (SAC), we focus on uninterrupted and reasonably safe autonomous driving without steering off the track for a considerable driving distance. Our proposed technique exhibits learning in autonomous vehicles under complex interactions with the environment, without being explicitly trained on driving datasets. To ensure the effectiveness of the scheme over a sustained period of time, we employ a reward-penalty based system where a negative reward is associated with an unfavourable action and a positive reward is awarded for favourable actions. The results obtained through simulations on DonKey simulator show the effectiveness of our proposed method by examining the variations in policy loss, value loss, reward function, and cumulative reward for ‘VAE+DDPG’ and ‘VAE+SAC’ over the learning process.

## 1. Introduction

Self-driving cars, also known as autonomous vehicles (AV), driverless cars, smart transportation robots (STR) or robocars have a potential to change the way we commute [1]. Autonomous vehicles incorporate integration with digital infrastructure and smart cities and form a critical component of the connected and autonomous vehicles (CAV) and internet of vehicles (IoV) framework [1].

A long-standing goal of artificial intelligence (AI) has been to drive a vehicle in a safe manner [2]. Recent advances in deep learning (DL) and state representation learning (SRL) have intensified research to develop autonomous agents with human-level capabilities [2]. Deep reinforcement learning (DRL), a combination of DL and reinforcement learning (RL), has been widely used as a baseline format for the self-driving vehicles [1]. This has led to a surge in research activities to achieve the quality and the speed needed to simulate, test, and run autonomous vehicles using various DL paradigms.

With ubiquitous availability of cloud based processors such as Google Cloud Platform (GCP) and Amazon Web Services’ (AWS) Elastic Compute-2 (EC2), the complex process of fine-tuning and optimizing neural network architectures in SRL has been extensively simplified [3]. An enhanced and robust representation of driving environment leverages variational inference, stochastic gradient descent, and variational Bayes encoding in partially/completely observable driving environment [3]. Furthermore, SRL is imperative for stabilizing long-term driving behavior in autonomous vehicles as DRL based transition models may lead to divergence of the Markov states under complex Markovian transitions [4]. Assuming that the vehicle state-transitions may vary over each time-stamp, variational autoencoder (VAE) is used to learn the mappings in the driving environment followed by deep deterministic policy gradient (DDPG), and soft actor-critic (SAC) for inferring the latent state-action-reward tuples [4]. The state transition variation over each time stamp refers to transitions in vehicle states, modeled according to Markov Decision Process (MDP, introduced in Section 4). Each time-stamp refers to the duration of driving episode that is studied, before moving to next episode. The different timestamps also enable to find those state-action pairs that lead to abrupt variations, without affecting the continuous state-space of MDP.

In this paper, VAE+DDPG and VAE+SAC are proposed that combine DRL with SRL by mapping an input state vector to an action, solving the autonomous driving task. Simulation results show that the VAE+DDPG and VAE+SAC can learn reasonable continuous control policies from high-dimensional observations that contain robust task-relevant information. One of the earliest works in applying DRL to autonomous driving has been presented in Reference [5]. This work was enhanced by Reference [6] where some of the instabilities in policy training encountered by Reference [5] were addressed using VAE. Our work builds on the lines of these works and compares the performance of VAE+DDPG and VAE+SAC in autonomous driving.

Deep reinforcement learning has been widely applied to various problems, predominantly in game playing [7,8]. Deep reinforcement learning has also been extensively applied to resource allocation and channel estimation problems in wireless communication, autonomous routing and self-healing in networking, localization and path-planning in unmanned air vehicles (UAV), smart-drones and underwater communications. As wireless networks and applications become more decentralized and autonomous, the network nodes are required to make decisions locally in order to maximize the system performance in a dynamic and uncertain environment. Obtaining an optimal policy in reasonable time, taking decisions and actions under large state-spaces using DRL have been applied to network access, wireless caching, cognitive spectrum sensing, and network security. Some of the more recent DRL applications include modeling multiple experience pools for UAV autonomous motion planning in complex unknown environments [9], learning output reference model tracking for higher-order nonlinear systems with unknown dynamics [10], and pick and place operations in logistics using a mobile manipulator controlled with DRL [11]. The DRL paradigm has been extended to domains such as autonomous vehicles and has opened new research avenues [12]. Model-free DRL technique known as Q-learning offers a compelling technique to further explore the problem of autonomous driving without explicitly modelling the driving environment [13]. The paper explores the role of SRL to complement the autonomous driving problem modeled using an MDP. The results provide a significant groundwork for considering solutions to some autonomous driving problems using standalone SRL-DRL framework. The current work considers a simple driving environment, consisting of a single-lane straight-line driving trajectory. There are no obstacles on the driving path and the roads are devoid of any sharp turns, bends, and curvatures. In the future, the driving scenario is expected to emulate more real-world driving complexities, extended to multiple vehicles in the driving lane, vehicles on the oncoming lane, traffic lights, intersections, and curvatures in the driving trajectory. Our results outperform some data-driven models based on the accuracy, maximum error-free drive-time before deviating off the track and policy losses [14]. The state-of-the-art developments in DL and RL were the principal motivation behind this work. The contributions of this paper are summarized as follows:

- Proposing a SRL based solution using VAE, SAC, and DDPG to solve the autonomous driving problem formulated as a MDP. We propose a SRL algorithm that combines the feature extraction capabilities with the fast and powerful batch RL approach of VAE [15].
- Analysis of the autonomous driving behavior, verification of the proposed SRL-DRL scheme, and performance comparison of VAE+DDPG and VAE+SAC approaches. This is done by the implementation of VAE, DDPG, and SAC based autonomous driving in DonKey simulator [16,17], that closely approximates real-world driving conditions.

The rest of this paper is organized as follows—in Section 2, a brief literature review on the topic of autonomous vehicles and application of current deep learning technologies to autonomous vehicles is presented. In Section 3, the system model is introduced. The problem formulation describing autonomous driving in continuous state-space as MDP and proposed SRL solution approaches comprising VAE+DDPG and VAE+SAC are presented in Section 4. The proposed solution approach based on MDP and Bellman optimality equations is presented in Section 5. The experimental setup in DonKey simulator to capture real-time driving images for VAE pre-processing to select robust features is presented in Section 6. Simulation results and discussions are elaborated in Section 7 with a comparison of performance characteristics of VAE+DDPG and VAE+SAC approaches. Finally, Section 8 provides concluding remarks and some directions for future work.

## 2. Literature Review

### 2.1. Recent Advances and Bottlenecks in Realizing Self-Driving Vehicles

Analysis of the behavior and strategies frequently employed by safe human drivers is essential in the development of AV [18]. Considerable research has gone into extracting the driving strategies adopted by drivers and to model their behavior without human intervention in varying situations [18]. In the last decade, machine learning (ML) algorithms such as fuzzy logic control, predictive and adaptive control, hybrid dynamical models and Markov chain models have been used to model the driving behavior. The predictive control based theories successfully predicted and reproduced human driving behavior under constrained environments [19]. However, the method relied heavily on data collection and representation as prior knowledge about the rule base was essential. The accuracy of Markov chain models depends on the prior knowledge about the state transition probability between each state pairs [15]. In realistic scenarios, the diverse driving conditions to be encountered are unlikely to be entirely known in advance. With rapid breakthroughs in DL, attempts to learn driving behavior directly from the driving data without prior knowledge of the driving conditions have gained prominence in recent years [3].

Deep learning techniques have gained increased attention as their design requires minimal prior knowledge and the models can be fine-tuned to scale to different environments [20]. These models have been enhanced using recurrent neural networks (RNN) that memorize long-term dependencies and tackle autonomous driving as partially observable Markov decision processes (POMDP) [21]. This is a significant improvement over traditional methods such as Bayesian decision process based on Markov assumption [22]. The POMDPs formulate the autonomous vehicle control problem as an optimization task, and rely on assumptions to optimize an objective [21]. The RL seems to be promising for planning and control aspects and scales to very complex environments and unexpected scenarios [2].

Recently, researchers have tried to divide the autonomous vehicle problem into sub-problems for categories such as object-detection, scene-segmentation, visual odometry and combine the results together [1]. However, the sub-problems might be more complicated than the autonomous driving task itself. For example, object detection using single shot multibox detector (SSD) in driving environment is redundant, as human drivers do not detect and classifiy all the objects; rather, they classify the most relevant objects [23]. Moreover, the motion of the vehicle introduces a Doppler shift which causes dynamic contraction of the visual zone [24]. Furthermore, the solutions to the isolated sub-problems could be optimum, fine-tuned and well-solved, but might not integrate so as to result in a cohrerent solution [25].

Deep reinforcement learning addresses these issues by introducing a reward signal that correlates current driving and future planning to arrive at optimum driving [6]. The reward is positive for a correct/favourable action, and negative for an incorrect/unfavourable/disastrous action [26]. As the reward involves driving manoeuvres frequently encountered in a real-time driving environment, training a DRL system on a simulated vehicle acts as a bridge to study the implications in real-world scenarios [24].

### 2.2. State Representation Learning and Deep Reinforcement Learning

As a combination of DL and RL, DRL does not require prior training on labelled data as in supervised learning, and tries to learn an optimal strategy from the environment through repeated corrective actions [23]. Based on deep Q-networks (DQN), considerable advances and breakthroughs have been achieved in the domain of gaming [21]. Many gaming applications utilize discrete asynchronous advance-actor critic (A3C) to train the agent [27]. However, it is impractical to switch steering, adjust speed, or take braking actions in binary levels comprising of two discrete values [2]. Realistically, the outcomes depending on continuous actions are more applicable to driving scenarios [28].

**Figure 1:**
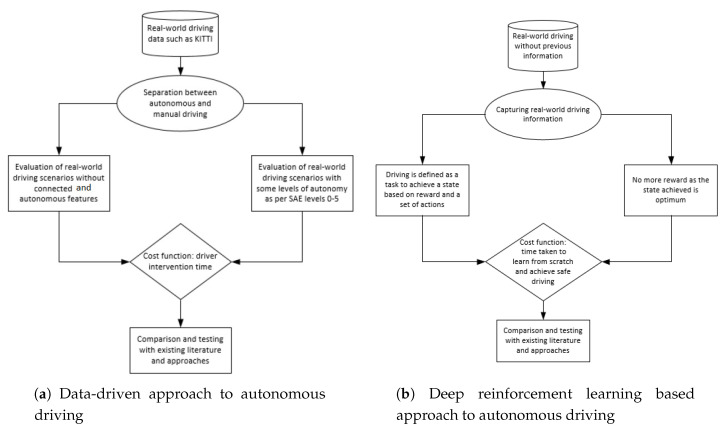
A comparison of data-driven and deep reinforcement learning based approaches to autonomous driving [31,32].

Deep reinforcement learning provides the ability to output continuous action using DDPG and SAC for driving behavior [3]. Both DDPG and SAC can be further enhanced when instead of being trained on raw input, they are trained on outputs obtained through VAE with pre-defined loss functions [29]. For instance, the steering actions in a vehicle fluctuate a lot when the agent is trying to maintain its position in a lane or while making a turn [30]. In this paper, we investigate the applicability of VAE, DDPG, and SAC for autonomous driving. Moreover, we show that our approach using VAE, SAC, and DDPG reduces the learning time and leads to longer episodes of uninterrupted driving. As the existing inference frameworks pose challenges for learning optimal policies, the application of probabilistic models to RL leads to optimization through variational inference [27]. A brief comparison of data-driven and DRL based approaches to autonomous driving is depicted in Figure 1.

**Figure 2:**
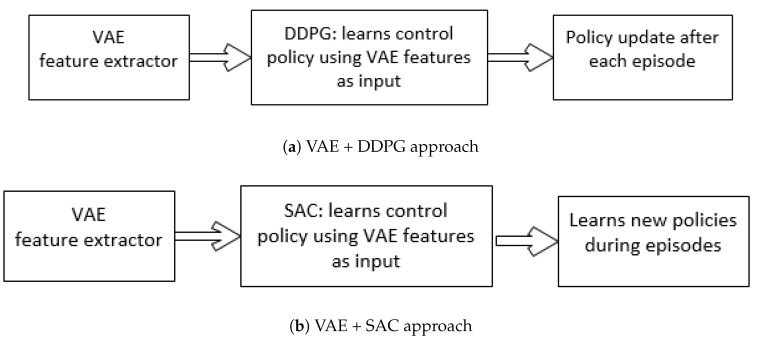
Block diagrams representing variational autoencoder (VAE) + deep deterministic policy gradient (DDPG) and VAE + soft actor-critic (SAC) approaches.

In the existing literature, the driving environment is usually Rayleigh distributed [8]. Actor-critic methods have achieved incredible performance on RL problems such as games, but they are prone to instability due to frequent interaction between the actor and critic during learning [7]. An inaccurate step taken at one stage might adversely affect the subsequent steps, destabilizing the learning. To avoid such issues, rewards were introduced to regularize the learning objective of the actor by penalizing the error of the critic [33]. This improves stability, as large steps in the actor update are prevented when the critic is inaccurate [27]. A brief comparison of VAE+DDPG and VAE+SAC techniques is depicted in Figure 2.

## 3. System Model

Let $X_{t}$ represent the image dataset, $X_{t}$ = $\left\{x_{t-n+1},x_{t-n+2},...,x_{t}\right\}$ where $x_{t}$ denotes the *t*-th frame image in the dataset consisting *n* frames associated with states of the vehicle at a given time, given by $S_{t}=\left\{s_{t-n+1},s_{t-n+2},...,s_{t}\right\}$, and the actions taken $A_{t}=\left\{a_{t-n+1},a_{t-n+2},...,a_{t}\right\}$ based on the features learnt by the VAE feature extractor [21]. In order to analyze the different positions of the vehicle in a given time-stamp, the vehicle while trying to maximize the reward function seeks to execute certain actions. The actions can be to accelerate, to decelerate, maintain the same velocity, to turn right, turn left, or continue in the same direction [21]. Given that the vehicle transition from one state to another is often a continual process, a MDP is proposed in this paper to model the vehicle transitions [1].

**Figure 3:**
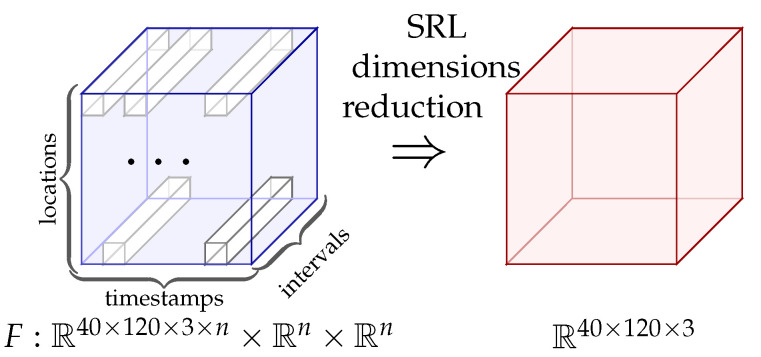
State representation learning (SRL) dimensionality reduction.

**Figure 4:**
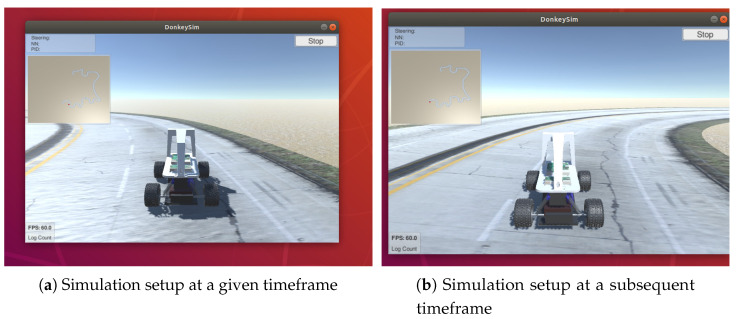
Driving manoeuvres in generated road driving environment in Donkey simulator at a given timeframe [16].

Learning to maintain a straight line path on the road can be defined as estimating the function $F:R^{40\times 120\times 3\times n}\times R^{n}\times R^{n}\to R^{40\times 120\times 3}$ that identifies the Markovian states and predicts $x_{t+1}=F\left(X_{t},S_{t},A_{t}\right)$. Using SRL dimensionality reduction and robust feature retention, as depicted in Figure 3, the images captured by the autonomous driving agent in the driving environment are distributed into a Gaussian space, with the image output size represented by 40×120×3, where the dimensionality was chosen experimentally and the Gaussian assumption was in accordance with Bayes variational autoencoding [12]. The driving environment under consideration consists of a road, a navigating vehicle, and an obstacle (optional). A section of the driving scene with the road and vehicle is represented in Figure 4. At $x_{t+1}$ frame of the dataset, the vehicular agent determines its next possible set of *S* states depending on selecting an action from the set of *A* possible actions [34]. The function $F:R^{40\times 120\times 3\times n}\times R^{n}\times R^{n}\to R^{40\times 120\times 3}$ represents state-representation-learning approach, where driving environment images are down-sampled to only include more robust features, termed as SRL dimensionality reduction. The numbers 40×120 represent the scene size in 2 dimensions (2D), or the number of pixels in an image in 2D, experimentally chosen to retain sufficient image clarity, and multiplying by the number 3 indicates red, green, and blue (RGB) component of colors in the driving images [35,36]. The images in 3 dimensions (3D) are further downsampled to 2-dimensional (2D) images as this reduces processing power required while retaining robustness of features. The function *F* is learned in a piecewise manner so that the efficiency and performance can be improved separately [5]. The action values are continuously encoded as DDPG and VAE are suitable for continuous action spaces. In Section 5.2, Table 1, it is briefly described how these values continuously vary until vehicle stabilizes. Moreover, these values are seen to change depending on different initial reward functions, so the abstract values of these variables do not give a strong interpretation of vehicle behaviour, rather the vehicle behaviour is studied using parameters defined in Table 2.

### Performance Analysis

**Figure 5:**
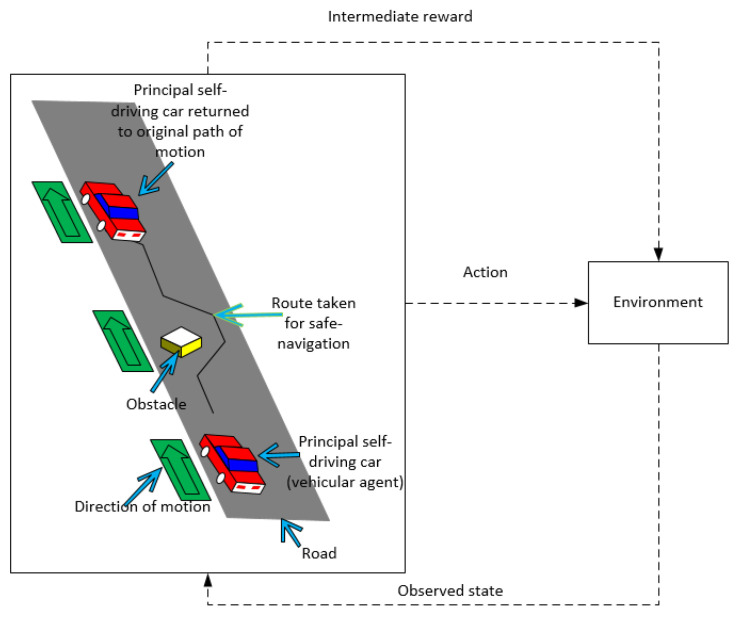
Deep reinforcement learning (DRL) for self-driving cars.

The system model is represented in Figure 5. In high dimensional dense spaces, the problem of action conditioned transitions and intermediate representations is important, because convergence probabilities and consequent control actions tend to become unstable at higher dimensions [4]. Moreover, highly nonlinear input data comprising spatio-temporal dependencies such as driving image sequences need to be iterated in large timesteps to achieve realistic long-term prediction. The goal is to obtain a model that best reflects a set of observed states [19,32].

## 4. Problem Formulation

The problems of autonomous driving scene perception, environment cognition, and decision making are investigated in this paper. The vehicle is represented as an agent which receives sensory inputs and performs driving actions (maneuvres) in an environment. The vehicular agent acts on rewards, penalties, and policy losses from the environment with the goal to maximize the rewards it receives, while minimizing the policy losses [37]. The agent learns an action based on the policy function, loss function and state-action-reward model. A state $S_{t}$ is Markov if and only if a future action is independent of the past actions and depends on the present state and actions [1]. We address the following issues pertaining to autonomous driving:

1. To generate realistic and safe autonomous driving using SRL-DRL and related techniques [22].
2. To improve the autonomous driving policies based on continuous state space, continuous action space, and pixel space [38].
3. To learn the driving behavior and environmental conditions without manual input, using SRL-DRL [33].
4. To reduce the inaccuracies, and improve loss function, entropy, policy, loss, and learning rate using improved VAE, DDPG, and SAE [27].
5. To maintain the trade-off between policy loss and learning rate [39].

An initial vehicle state can lead to a set of further movements and approaches to inform transitional probabilities of a Markov process over a driving state-space. Furthermore, the Markov process provides an assessment of the path through the driving environment with an expected policy loss function. When the autonomous vehicle adjusts its behaviour to make optimal decisions or to take actions that minimize an expected loss, the probability of the vehicle undergoing different state-action-reward tuples is given as:

(1) $$P\left[S_{t+1}|S_{t}\right]=P\left[S_{t+1}|S_{1},...,S_{t}\right],$$

where P defines the probability distribution of the vehicle state at a given timeframe. The state transition matrix *P* defines the transition probabilities from states *s* to successor states $s^{\prime }$ after taking action *a*. A MDP is defined as a tuple <S,A,P,R,γ> [34] where *S* is a finite set of states, *A* is a finite set of actions, *P* is a state transition probability matrix, *R* is a reward function, and γ is a discount factor, γ∈[0,1]. The reward function is a measure of entropy in state-action pair and it decides how well an action contributes to help an agent reach the best possible next state. The learning policy π is given as [24]:

(2) $$\begin{array}{c} \pi \left(a|s\right)=P\left[A_{t}=a|S_{t}=s\right]. \end{array}$$

The objective of the vehicle is to drive maximum possible distance on the road, staying in the lane without deviating off the track [12]. The driving action is terminated once the vehicle deviates off the track or trudges on the other lane, represented as

(3) $$\begin{array}{c} \min_{<V_{r},S>}V_{r}, \end{array}$$

where $V_{r}$ represents the parameter values, that is, the set of velocities in a given timestep, at present time and past instances for a given state *S*.

(4) $$\begin{array}{c} V_{r}=\left\{V_{r}\left(t_{0}\right),V_{r}\left(t_{1}\right),...V_{r}\left(t_{N-1}\right)\right\}, \end{array}$$

where *N* indicates the *N*th time frame. All past and present states are in the continuous state-space [34]. The parameter values, that is, the set of velocities in a given timestep, at present and past instances for a given state *S* are obtained for the trajectory followed by the vehicle described by [20,40]:

(5) $$\begin{array}{c} x_{c}\left(t_{i}\right)=x_{c}\left(t_{i-1}\right)+v_{x}\left(t_{i-1}\right)\Delta t+\left.\frac{1}{2}\right.v_{x}\left(t_{i-1}\right)-v_{x}\left(t_{i}\right)\Delta t,\forall i \end{array}$$

(6) $$\begin{array}{c} y_{c}\left(t_{i}\right)=y_{c}\left(t_{i-1}\right)+v_{y}\left(t_{i-1}\right)\Delta t+\left.\frac{1}{2}\right.v_{y}\left(t_{i-1}\right)-v_{y}\left(t_{i}\right)\Delta t,\forall i,\\ i=0,1,2.....N-1,t_{i}=i\Delta t, \end{array}$$

where Δt is the difference between two subsequent timeframes while the vehicle navigates the trajectory. These parameters are used to calculate the optimal value function $v_{\pi }^{*}\left(s\right)$ and optimal Q-value $q_{\pi }^{*}\left(s,a\right)$. In SRL, a reward function directly influences the behavior adopted by an an agent. Reward function refers to the feedback obtained from the environment to evaluate the viability of the actions taken. In autonomous driving, a reward function is formulated as a linear model based on the velocity of the car *v*, the angle between the road and car’s heading θ, and the distance from the middle of the road *d*. The reward *r*, given by (7) prevents the vehicle from deviating off the track while allowing the vehicle to maintain its position on the road [24].

(7) r=v(cosθ−d).

We propose an extra penalty as Ψ *$|s_{t+1}-s_{t}|$, where $s_{t}$ represents the state at time step *t* arrived due to action *a* and Ψ is the corresponding constant empirical coefficient. The experimental results show better smoothness with the new penalty and the whole reward function is given in (8) as [24]:

(8) $$r=v(\cos\theta -d-\Psi *|s_{t+1}-s_{t}|).$$

We set Ψ to 2 or 3 as driving smoothness tends to reduce with increase in throttle value. This fact conforms to human driving behavior where the faster the car runs, the harder it is to control it, indicated by failure to recognize turn/curve at high speed.

Although there may be more than one optimal policy, in autonomous driving situations, it is imperative to determine if there exists at least one optimal policy for a specific driving environment. The optimal state value function is given as [2]:

(9) $$\begin{array}{c} V^{*}\left(s\right)=\max_{\pi }V^{\pi }\left(s\right)\;,\forall s. \end{array}$$

In this paper, the autonomous driving problem is formulated as a MDP, on the lines of the work of Reference [5]. The MDP is then solved using policy gradient mechanism DDPG and actor-critic mechanism SAC, accompanied by VAE at initial solution stages [6]. The following questions pertaining to scene perception, environment cognition, and decision making are investigated in this paper:

1. To generate realistic and safe autonomous driving using DRL and related techniques [30].
2. To improve the autonomous driving policies based on continuous state space, continuous action space, and pixel space [12].
3. To learn the driving behavior and environmental conditions without manual input, using DRL [37].
4. To solve the inaccuracies, improving loss function, entropy, policy, loss, and learning rate using improved VAE, DDPG, and SAE [31].
5. To maintain the trade-off between policy loss and learning rate [41].

## 5. Proposed Solution

**Figure 6:**
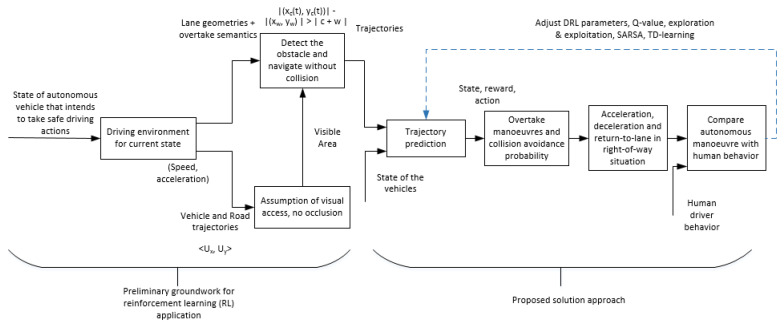
Proposed solution approach.

Autonomous driving is modelled as a multi-objective control problem with high-dimensional feature space, agent (vehicle) states, and a mono-dimensional discrete action space [32]. We use VAE to map the vehicle state at a given time and the dynamics of the environment not directly influenced by the vehicle. We repeat this procedure iteratively in a semi-batch approach to bootstrap the algorithm, starting from a fully random exploration of the driving environment. Beginning randomly, the vehicle is trained to learn how to take better decisions over repeated attempts, reducing errors based on a reward function [24,42]. The proposed solution using VAE, DDPG, and SAC aims to use reward-function to learn policies for multiple state-action-reward tuples. The solution enables the optimal policy to generalize the continuous actions for states that are yet to be traversed by the vehicular agent. The proposed solution is represented in Figure 6.

- Let’s say the vehicular agent has a choice of taking one of *k* possible actions $a_{1}\ldots a_{k}$.
- Assume that the environment can be in one of *m* different states $s_{1},\ldots ,s_{m}$.
- Upon taking an action $a_{i}$ in the environment in state $s_{j}$ the vehicle incurs a loss $\ell_{ij}$.
- Given the observed data *D* and prior background knowledge *B*, the vehicular agent’s beliefs about the state of the driving environment are denoted by p(s|D,B).
- The optimal action is the one which is expected to minimize loss and maximize utility.

After every action, the vehicle transitions from one state to another. The initial state of an autonomous vehicle is then updated to best adapt to the current state of the driving environment. This is done through constant adjustment of acceleration (and consequently speed) so that the vehicle trajectory is aligned with the road/lane trajectory. The alignment of trajectories for extended timeframes of vehicle navigation is an indicator that the vehicle is able to follow a lane and does not deviate off the track. To simplify the problem, we assume that the vehicle has an unobstructed and unoccluded visual access to the driving environment where any curves, turns, and obstacles in the vicinity are clearly visible to the vehicle. To ameliorate the problem complexity, and to overcome non-uniform, skewed, or intractable image distributions, we implemented learning action-prediction with a policy gradient architecture, (DDPG) and an actor-critic architecture, (SAC) both preceded by a variational auto encoder (VAE). The DRL approaches used are:

- VAE for efficient driving environment analysis,
- DDPG for optimal policy calculation and,
- SAC for faster arrival at optimal policy, without having to repeatedly train the DRL network for all the timeframes.

### 5.1. Solution Approach

This paper implements a feature extractor, VAE, to compress the images captured during driving to a lower dimensional space [23]. The weights that provide gradients with similar magnitudes indicate that each feature has been kept relevant. The first step of extracting the relevant information from raw data is done by VAE by compressing the search space. This also accelerates the training in later stages by learning the control policy from the lower dimensional image space [4,15].

The second step after the features have been extracted is to use a DRL algorithm. This paper investigates DDPG and SAC algorithms. The DDPG policy gradient algorithm learns a control policy using VAE features as input and the policy is updated after each episode [30]. A distinguishing feature offered by DDPG is the replay buffer, which is memory to store the interactions with the environment. These interactions can be played when needed at a later time, so that the self-driving car can update the policy without explicitly interacting with the environment in real-time again [32].

In our experiments, the vehicle is trained to maximize the distance travelled before it steers off the track. The episode ends as soon as the vehicle steers off the road. The episode termination also prevents the vehicular agent from exploring regions that do not contribute at all to effectively learn the driving task [40]. If a VAE feature extractor is trained after each episode, the distribution of features is not stationary. As the features change over time, this introduces instabilities in the learning policy [28]. Moreover, on low power CPU machines, traning a VAE after each episode is time consuming and a slow process. To address these issues, in this work, a VAE is pre-trained and a fixed set of features are collected beforehand [1]. Next, these features are provided as input to DDPG to learn and update the policy. Also, to speed up the process, we trained feature extractors using Google Colab notebook. Lastly, the DDPG algorithm is known to be inherently unstable in cases where its performance degrades during training and fails to tune if there are multiple factors that affect the learning outcome [23]. The SAC algorithm that provides much stable performance and is easier to tune in case of multiple parameters is applied and its performance comparison with DDPG is analyzed.

### 5.2. Solving MDPs Using Bellman Expectation Equations

The policy gradient DRL methods and the actor-critic methods can be solved using Bellman equations mentioned below. The parameterized policy defines how the autonomous vehicle selects its actions and the critic appraises each action taken by the vehicular agent in the driving environment. The appraisal is associated with a positive or negative reward function according to which the parameters of the actor are updated [24]. Similarly, the actor’s parameters can be updated with a policy gradient that does not necessarily have a critic component [1]. The policy gradient methods such as DDPG adjust the policy parameters based on a sampled reward measured against a baseline value [28]. In DDPG, this baseline is a stationary value that does not update with experience. In SAC, a baseline is estimated from experience, making the method an actor-critic method where the vehicle updates its parameters after each step taken in the driving environment [40]. The Bellman equations allow comparing the results of taking a different action in each state and assist in negating the wrong step, causing the agent to strengthen how it selects the apparent best action. In SAC, the critic components make the gradient point in the apparent best direction without sampling other actions [24].

(10) $$\begin{array}{c} v_{\pi }\left(s\right)=\sum_{a\in A(s)}\pi \left(a|s\right)\sum_{s^{\prime }\in S,r\in R}p\left(s^{\prime },r|s,a\right)\left(r+\gamma v_{\pi }\left(s^{\prime }\right)\right), \end{array}$$

(11) $$\begin{array}{c} q_{\pi }\left(s,a\right)=\sum_{s^{\prime }\in S,r\in R}p\left(s^{\prime },r|s,a\right)\left(r+\gamma \sum_{a^{\prime }\in A\left(s^{\prime }\right)}\pi \left(a^{\prime }|s^{\prime }\right)q_{\pi }\left(s^{\prime },a^{\prime }\right)\right). \end{array}$$

The Bellman optimality equation is given as [5]:

(12) $$\begin{array}{c} v_{\pi }^{*}\left(s\right)=\max_{a\in A(s)}\sum_{s^{\prime }\in S,r\in R}p\left(s^{\prime },r|s,a\right)\left(r+\gamma v_{*}\left(s^{\prime }\right)\right) \end{array}$$

(13) $$\begin{array}{c} q_{{*}_{\pi }}\left(s,a\right)=\sum_{s^{\prime }\in S,r\in R}p\left(s^{\prime },r|s,a\right)\left(r+\gamma \max_{a^{\prime }\in A\left(s^{\prime }\right)}q_{*}\left(s^{\prime },a^{\prime }\right)\right). \end{array}$$

Given the optimal value function, $V^{*}$, a popular technique to get optimal policy $\pi^{*}$ is the greedy algorithm. This algorithm specifies that for a vehicle in a current state *S* and an optimal value function $V^{*}$, the actions *A* that appear best after a one-step search will be optimal. The greedy algorithm makes a choice to select an action that seems to be the best at that state, making a locally-optimal choice and verifying its suitability as a globally-optimal solution. $V^{*}$ turns a long-term reward into a measurable quantity which is locally and immediately available. $Q^{*}$ is used to get the optimal policy [5]. The policy improvement theorem justifies an optimal action in a given state [43]. In Equations (14) and (15), the vehicular agent is characterized by policy $\pi^{*}\left(s,a\right)$, $V^{\pi }\left(s\right)$ describes how good it is for a vehicle to be in a given state *s* depending on policy $\pi^{*}\left(s,a\right)$, and $Q^{\pi }\left(s,\pi^{\prime }\left(s\right)\right)$ describes how good it is for a vehicle to choose an action *a* after being in a given state *s*.

(14) $$\begin{array}{c} \pi^{*}\left(s,a\right)=0\;\;\forall a\;\;s.t.\;\;Q^{*}\left(s,a\right)\neq \max_{a^{\prime }}Q^{*}\left(s,a^{\prime }\right), \end{array}$$

(15) $$\begin{array}{c} Q^{\pi }\left(s,\pi^{\prime }\left(s\right)\right)\;\geq \;V^{\pi }\left(s\right)\;\forall s\;\;⟹\;\;V^{\pi^{\prime }}\left(s\right)\;\geq \;V^{\pi }\left(s\right). \end{array}$$

The vehicle at a given time in a given state has two possible actions to choose from:

1. $ac_{1}$: accelerate, and
2. $ac_{2}$: don’t accelerate.

In an ideal scenario, if acceleration would lead to an unsafe action such as drifting off the track, or deviating on to the other lane intended for oncoming vehicles, there are two probable outcomes:

1. $s_{1}^{d}$: safe driving, and
2. $s_{2}^{d}$: unsafe driving.

The vehicular agent needs to arrive at an optimal action for this decision problem. Based on MDP, the following variables are defined for the vehicular agent:

- States: $s_{t}$
- Actions: $a_{t}$
- Rewards: $r_{t}$

The variable $s_{t}$ defines the state of the driving environment and the vehicular agent at time *t*. The vehicular agent takes action $a_{t}$ and receives reward or penalty $r_{t}$. The reward is assumed to depend on the state and the action. The optimal policy is defined by the Equation (16).

(16) $$\pi^{*}=\underset{\pi }{\arg\max}E\left[R_{t}|\pi \right].$$

The initial values for states and the transition probabilities are depicted in Table 1. For a continuous dynamical driving environment, the initial probability components are non-negative entries and sum up to 1. If $P(s_{1}^{d}|ac_{1})$ is a probability that represents the initial safe driving state of the vehicle, then as per the Markov property of Equations (1) and (16), the subsequent components in Table 1 represent the probability that the vehicle maintains the safe state. The transition probabilities emphasize the variations in reward function. The driving behaviour might show abrupt variations in driving due to changes that represent transitions that occur between subsequent states. Detection of abrupt change points is useful in modeling and predicting driving behavior and the transition probabilities enumerate, categorize, and compare the reward values. Under safe driving condition, if the probability of acceleration is very high, then the loss is set to a high negative value, indicating a positive reward for that action. Under unsafe driving condition, if the vehicle decides to accelerate, the associated loss is a positive value, indicating negative reward, thus prompting the vehicle to refrain from taking the accelerating action. Similarly, if the vehicle is driving in a safe state and decides not to accelerate, the reward remains neutral, indicated by zero loss. Vice versa, if the vehicle is in an unsafe state and decides not to accelerate, although the action will not deteriorate the vehicle’s state further, it will not guarantee a return to safe driving either. This is implied through a zero reward. The appraisal is associated with a positive or negative reward function according to which the parameters of the actor are updated. Similarly, the actor’s parameters can be updated with a policy gradient that does not necessarily have a critic component.

## 6. Experimental Setup

In this paper, the experiments are carried out on the Ubuntu operating system, DonKey simulator, OpenAIgym, and Google Collaboratory [16,17]. Due to the property of learning by trial and error in SRL, simulator plays an important role. Among various car simulators, the open racing car simulator (TORCS) is widely used, providing both front view images and extracted features. TORCS requires less hardware performance whereas DonKey provides flexibility and realism, hence we selected DonKey [16,17]. The raw sensor-data and the camera frames files are easy to use in DonKey simulator with the help of Docker, a container management software.

In DonKey simulator, driving environment consists of a road with two lanes, differentiated with a lane marker. Although the road curvature needs the vehicle to make a slight change in steering angle to stay on the road, the road is devoid of any sharp turns and the major part of the trajectory is linear. The specific inputs are gleaned as robust features from driving scenario mentioned and depicted in Figure 4, and have been considered in Section 4. To stay on the road, the rewards for action at a specific state are supposed to be high for preferable actions. However, once the vehicle is close to achieving good position in the lane, the reward for next action is considerably lower than in the beginning. In the simulation time over seven hours of driving, the captured frames are 160×320 pixels in the region depicting the vicinity of the middle of the road/lane.

We focus on the acceleration (speed), policy entropy, policy loss, and total timesteps. The acceleration is considered in x and y directions, so that maintains the vehicle velocity and position along x and y directions. Moreover, the acceleration along the y-axis takes the vehicle forward, whereas acceleration along the x-axis aligns the vehicle on the lane. We also record the timestamps at which these parameters were measured and the timestamps the camera frames were captured. We pre-processed the camera frames by downsampling them to 40×120 and normalizing the pixel values between −1 and 1. The self-driving vehicle needs to learn the road images, and a VAE encodes the road images into probabilistic Gaussian space as well as decodes them to 3D space. DDPG receives random pixel samples as input from the Gaussian distributed latent space and output of the VAE network. We trained the VAE for 200 epochs. Each epoch consisted of 10,000 gradient updates with a batch size of 64. The parameter fine tuning was done during programming the scenarios, using tune.run() argument and the optimal policy-loss values are obtained by hyper-parameter tuning. The DRL approach is slightly advantageous compared to convolutional neural networks (CNN) based approach as the need for explicit hyper-parameter tuning beforehand is bypassed.

## 7. Simulation Results

The simulation results are presented to show the performance of the proposed VAE+DDPG and VAE+SAC schemes. First, the simulation parameters are introduced followed by the simulation results. Next, the rewards and penalties for DDPG and SAC approaches are compared for driving environment. Then, the performance of the proposed approach is studied in terms of learning rate and acquiring stable driving state. Table 2 highlights the features and parameters considered in the simulation.

### 7.1. Performance Analysis

This section provides comparison of vehicle control algorithms using SRL based on policy gradients (VAE+DDPG) and actor-critic (VAE+SAC). The section discusses the results highlighting the probability of the autonomous vehicle transitioning into a new state, based on the cumulative reward achieved at each timeframe and the execution of an action. Since the autonomous driving problem is defined as first-order Markov decision problem, the state of the vehicle at next timeframe is dependent only on the present state and not the past states. This section compares the viability of VAE+DDPG and VAE+SAC approaches in ensuring that the autonomous vehicle arrives at the current state. The variations in cumulative reward accumulated over each timeframe in the presence of possible states in the VAE encoded driving environment is also discussed in this section.

#### Simulation Parameters

The parameters under consideration in this paper are described briefly as follows:

- Cumulative Reward: It describes the mean cumulative episode reward over all the states of the agent over a specific timestamp. The value usually increases during a successful training session. The general trend in reward is to consistently increase over time with some small ups and downs based on the complexity of the task. However, a significant increase in reward may not be apparent until the training process has undergone multiple iterations [44,45].
- Entropy: It is a measure of how random the decisions of the autonomous agent are. It should gradually decrease during a successful training process. In case the entropy decreases too quickly or does not decrease at all, the DRL architecture’s hyper-parameters are reset to a different initial value both in continuous as well as discrete action space [44,45].
- Episode Length: The mean length of each episode in the driving environment for the autonomous vehicular agent [44,45].
- Learning Rate: It signifies the step size taken at a time by the training algorithm to search for the optimal policy [44,45].
- Policy Loss: The policy is defined as the process for deciding actions that lead to optimal driving in the given scenario. Policy loss describes the mean magnitude of policy loss function. This loss correlates to how much the policy changes during an episode in a given timeframe [44,45].
- Value Estimate: It is the mean value estimate for all states visited by the autonomous agent. It corresponds to how much future reward the agent expects to receive at any given state [44,45].
- Value Loss: It defines the mean loss of the value function update. It correlates to how well the model is able to predict the value of each state. This should increase while the agent is learning, and then decrease once the reward stabilizes. These values also increase as the reward increases, and then decrease as the reward tends to becomes stable [44,45].

### 7.2. Rewards vs. Timesteps

**Figure 7:**
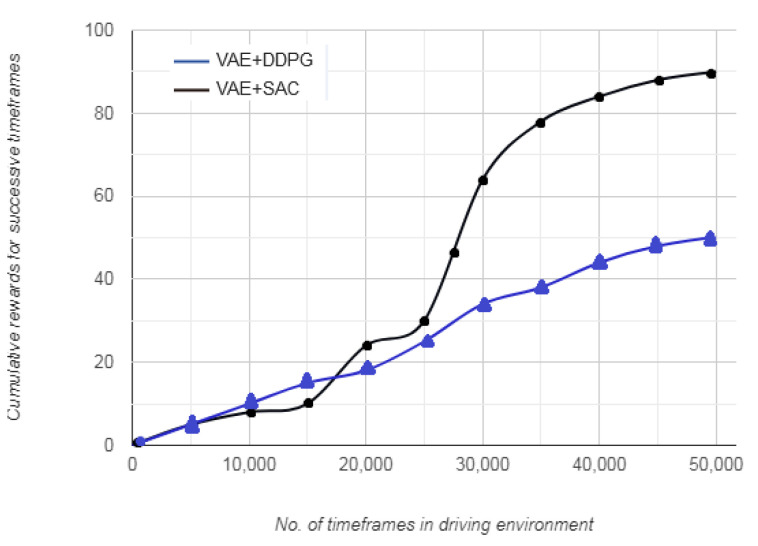
Cumulative reward vs. no. of timeframes.

Figure 7 represents the variation in cumulative reward with number of timesteps covererd by the vehicle in the simulated driving environment. For the first 20,000 timeframes, the cumulative reward gradually increases. This gradual increase indicates that the vehicle begins with a randomly defined initial state *S*, and selects a random action *A* with an aim to maintain its position on the lane. After initial haphazard movements, from 20,000–40,000 timeframes, the cumulative reward continues to increase at a similar rate for VAE+SAC approach, whereas the increase is steeper for VAE+DDPG approach. Furthermore, the VAE+DDPG approach shows minor fluctuations in a specific range, from 10,000–25,000 timeframes. This indicates that as the vehicle learns an optimum action, the deviation from those set of actions attracts a higher penalty as compared to that in the beginning. Consequently, the reward for successive favorable set of actions is less and indicates that the vehicle has to process a smaller set of data to arrive at that action, resulting in nearly smooth cumulative reward.

**Figure 8:**
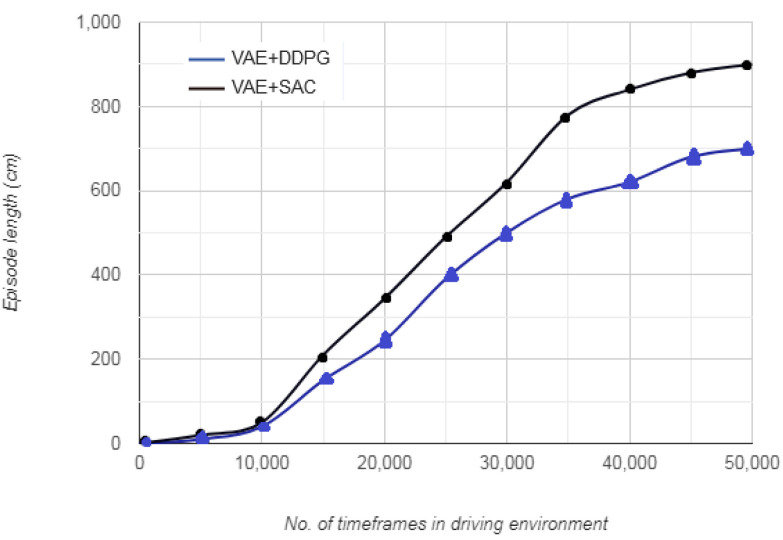
Episode length vs. no. of timeframes.

Figure 8 represents the variations in episode length with the number of timeframes in the driving environment. The episode length defines how long the autonomous vehicle occupies the road before returning to the original position. For the first 10,000 timesteps, the episode length traversed by the vehicle is approximately 900 cm for VAE+SAC algorithm and 700 cm for VAE+DDPG algorithm. This period indicates random initial learning by the vehicle that results in a haphazard motion. After the iterations in the first 10,000 timesteps, the episode length traversed by the vehicle begins to constantly increase. After 40,000 timesteps, the episode length does not show a large increase for both VAE+DDPG and VAE+SAC algorithms. This is congruous to the pattern followed by cumulative reward, indicating that once the vehicle identifies a set of favourable actions, the vehicle is able to remain on the road for longer episode lengths, before resorting to terminating action.

**Figure 9:**
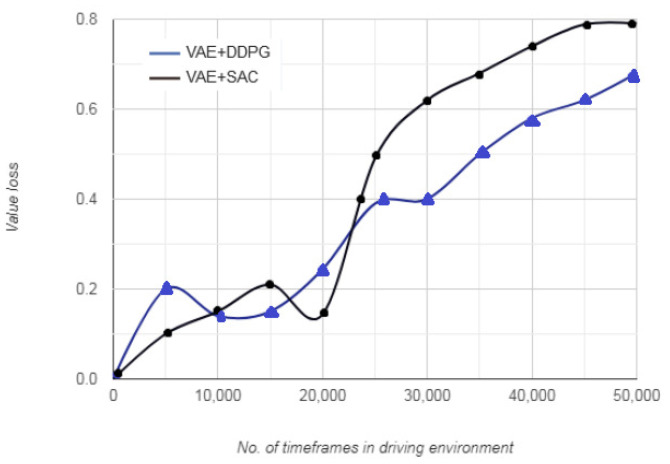
Value loss vs. no. of timeframes.

Figure 9 depicts the value loss versus the distance traversed by the vehicle before terminating an episode. For the first 10,000 timeframes, the value loss for each successive timestamp indicates that even when the reward function stabilizes, the value loss continues to increase in accordance with the time spent by the vehicle in the driving environment. This implies that at every new timeframe, the vehicle calculates a set of state-action-reward tuple to seek an optimum action. In addition, the value loss tends to exhibit lesser variations based on the multiple state-action-reward cycles that allow the vehicle to arrive at an optimal policy and learn future actions.

### 7.3. Learning Losses and Optimal Driving Policy

The losses describe the delay at arriving at optimal policy in VAE+DDPG and VAE+SAC. The policy losses indicate how much the policy is changing at each timestep with subsequent actions. During a successful learning phase, the vehicle after starting with random decisions must arrive at more coherent pattern of state, action, and reward.

**Figure 10:**
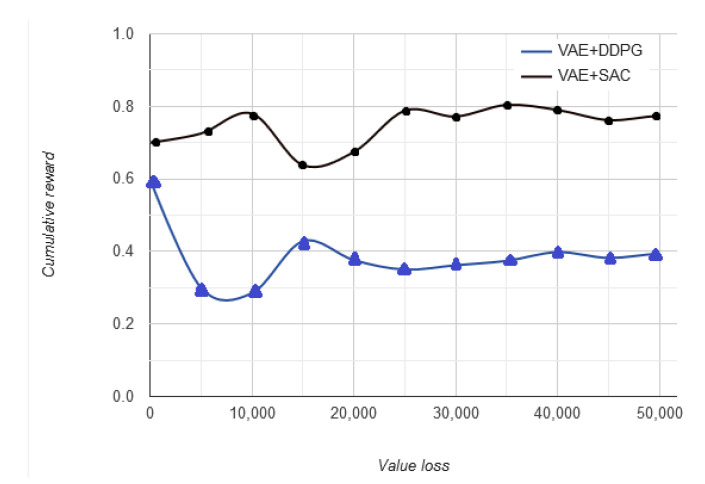
Cumulative reward vs. no. of timeframes.

Figure 10 highlights the fact that at the time of prediction of next state and to choose an appropriate action, the vehicle uses the cumulative reward in variation with policy loss to predict the next best action to take in the driving environment. The input state is the Q-values for all actions and the maximum cumulative reward for taking an action impacts the next reward predicted.

**Figure 11:**
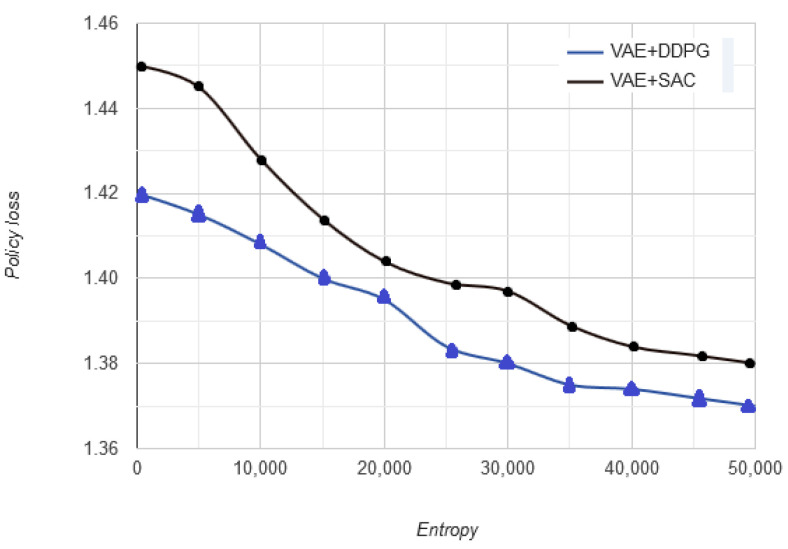
Policy loss vs. no. of timeframes.

The optimal driving policy indicates that the optimal action is taken at a given state. At a given state whether the action is optimal or not is plotted in policy loss vs. no. of timeframes as shown in Figure 11. As the vehicle approaches optimal decision, the randomness in decisions tends to decrease. During a successful scene understanding of the driving environment, decreasing randomness indicates that the vehicle has learnt optimally. The higher policy loss in the beginning indicates that as the vehicle moves in a haphazard direction, the algorithm traverses large number of states to adjust vehicle behavior. However, as the vehicle learns more about the driving environment, the haphazard motion is replaced by a more stable trajectory with less random movements, leading to gradually decreasing policy loss. In DDPG and SAC, these losses are defined and synthesized from unlabeled inputs (processed through VAE), and the variations in losses defined by the reward function.

### 7.4. Performance Comparison for VAE+DDPG vs. VAE+SAC

This subsection compares the performance characteristics of VAE+DDPG and VAE+SAC approaches for the autonomous vehicle to learn driving behavior. The vehicle arrives at an optimal state-value function $v_{\pi }^{*}$ after a specific timestep encompassing different iterations of function $F(X_{t},S_{t},A_{t})$ representing state-action tuple for the driving environment.

**Figure 12:**
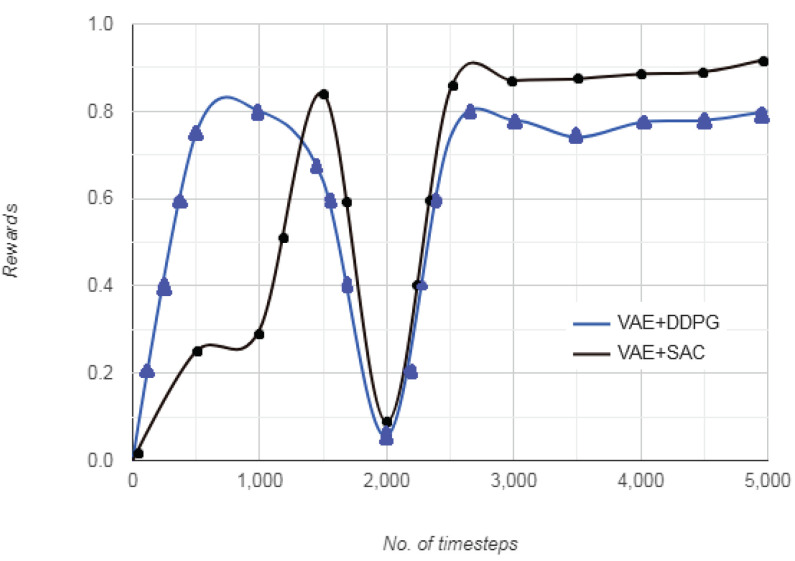
Comparison of rewards vs. number of training steps for VAE+DDPG and VAE+SAC.

Figure 12 represents the plot of rewards vs. the number of timesteps in the driving environment. As seen from the figure, for same number of timesteps, after the driving scenario images are processed through VAE, both DDPG and SAC converge after approximately 3000 timesteps. However, the initial randomness in vehicle motion is less in VAE+SAC as compared to VAE+DDPG. For upto 2000 timesteps during training phase, the vehicle depicts more haphazard movement to arrive at an optimum action for VAE+DDPG and settles comparatively quicker with VAE+SAC approach. The reward becomes constant after approximately 3000 timesteps, indicating that optimal value function $v_{\pi }^{*}$ and optimal state-action value function $q_{\pi }^{*}\left(s,a\right)$ has been approximated by the vehicular agent. The gradual increase indicates that the vehicle begins with a randomly defined initial state *S*, and selects a random action *A* with an aim to maintain its position on the lane. After initial movement, the cumulative reward continues to increase at a similar rate for VAE+SAC approach, whereas the increase is steeper for VAE+DDPG approach. Furthermore, as the vehicle learns an optimum action, the deviation from those set of actions attracts a higher penalty as compared to that in the beginning. Consequently, the reward for successive favorable set of actions is lesser and indicates that the vehicle has to process a smaller set of data to arrive at that action, resulting in nearly smooth cumulative reward.

The VAE+SAC eliminates the need to retain the state-action information until episode termination to compute value loss, being independent of cumulative rewards and other domain dynamics that might render driving environment representation challenging. The rewards represent the entropy of the learned policy giving an insight into how the vehicular agent learned to navigate the driving environment as well as managed to keep the episode length high. The autonomous vehicle agent has to choose from a set of possible actions, that is, accelerating, decelerating, or maintaining the same velocity. The reward for the first 1000 timesteps indicates that the vehicle proceeds with a random direction, so it has learned a policy with 0–80% probability of going haphazardly. From 1000–3000 timesteps, the rewards reorganize, indicating a reset of state-action-reward pair. After 3000 timesteps to 5000 timesteps, the rewards are almost stationary for both VAE+DDPG and VAE+SAC approaches. This indicates that the autonomous vehicle has learned the driving environment and has decided on the optimal action. Also, the reward is slightly higher for VAE+DDPG as compared to VAE+SAC.

## 8. Conclusions and Future Work

In this paper, we applied state representation learning to object detection and safe navigation while enhancing an autonomous vehicle’s ability to discern meaningful information from surrounding data. The proposed method used VAE, DDPG, and SAC to implement and analyze a combination of policy function, reward, and penalty to ensure that the autonomous vehicle stays on the track for maximum time in a given timeframe. In a particular driving state, based on the past instances of off-track deviations and episode terminations over several frames of previous iterations, the vehicle reinforces its behavior to maximize the reward function.

Applying SRL to autonomous driving has been proposed and implemented as an alternative approach to conventional DRL algorithms that require a large number of training samples for learning, which is infeasible and time-consuming in real-world driving scenarios. The application of VAE preprocessing enhanced the sample efficiency facilitating the learning process with fewer but robust samples. In this paper, capturing and interpreting the driving environment and possible set of actions a vehicle can take is effectively done using MDP for modeling the environment and generating complex distributions using VAE. The interpretation of the gathered data to execute meaningful action is done using policy gradient or actor critic based DRL methods.

The contribution of this paper is twofold. We proposed two DRL algorithms, VAE+DDPG and VAE+SAC. The combination of these techniques leads to smooth policy update in value function based DRL with enhanced capability of automatic feature extraction. Performing basic driving manoeuvres using non-DRL methods requires direct access to state variables as well as well-designed hand-engineered features extracted from sensory inputs. The DRL paradigm allows an autonomous vehicular agent to learn complex policies with high-dimensional observations as inputs. The driving environment images offer a suitable mechanism to learn to drive on a road in a manner similar to human driving.

Some future research directions are proposed below:

- To enhance the performance speed of the SRL approach used in this paper, the autonomous vehicle can be trained to learn a transition model in the embedded state-space using action conditioned RNN and long short term memory (LSTM).
- We aim to extend the VAE to learn the pixel space defined by Gaussian framework to generate realistic looking frames, images and videos predicting the autonomous vehicle behaviour. This would be a step forward towards receiving feedback-based corrective action ahead of the next timeframe.
- The driving environments can be subjected to real-world limitations, disturbances, and abrupt variations in operating conditions.
- For an autonomous vehicles to increase the uninterrupted drive-time, DRL techniques can be used in conjunction with probabilistic DL models to learn features from latent variables.

## Figures and Tables

**Table 1:** The initial values for states and transition probabilities.

$P(s_{1}^{d}|ac_{1})=0.99995$	$\ell_{11}=-10,000$
$P(s_{2}^{d}|ac_{1})=0.00005$	$\ell_{12}=+0.9$
$P(s_{1}^{d}|ac_{2})=0$	$\ell_{21}=0$
$P(s_{2}^{d}|ac_{2})=1$	$\ell_{22}=0$

**Table 2:** Features considered in the simulation.

Feature Name	Description
policy_entropy	An initial measure of randomness of vehicular agent’s decisions
policy_loss	Mean magnitude of Q-function, $q_{{*}_{\pi }}\left(s,a\right)$, calculated using Bellman optimality Equation (13)
serial_timesteps	Total timesteps for which image frames are sampled
time_elapsed	Time taken for vehicular agent to achieve stability and stay on the intended path
value_loss	The total loss function, $v_{\pi }^{*}\left(s\right)$, calculated using Bellman optimality Equation (12)
n_updates	The number of iterations of VAE+DDPG and VAE+SAC algorithm
